# Case Study of Ecstatic Meditation: fMRI and EEG Evidence of Self-Stimulating a Reward System

**DOI:** 10.1155/2013/653572

**Published:** 2013-05-02

**Authors:** Michael R. Hagerty, Julian Isaacs, Leigh Brasington, Larry Shupe, Eberhard E. Fetz, Steven C. Cramer

**Affiliations:** ^1^University of California, Davis and Wellspring Institute, Davis, CA 95616, USA; ^2^Wellspring Institute, San Rafael, CA 94903, USA; ^3^Barre Center for Buddhist Studies, Barre, MA 01005, USA; ^4^University of Washington, Seattle, WA 98195, USA; ^5^Physiology & Biophysics, University of Washington, Seattle, WA 98195, USA; ^6^Department of Neurology and Anatomy & Neurobiology, University of California, Irvine, CA 92697, USA

## Abstract

We report the first neural recording during ecstatic meditations called jhanas and test whether a brain reward system plays a role in the joy reported. Jhanas are Altered States of Consciousness (ASC) that imply major brain changes based on subjective reports: (1) external awareness dims, (2) internal verbalizations fade, (3) the sense of personal boundaries is altered, (4) attention is highly focused on the object of meditation, and (5) joy increases to high levels. The fMRI and EEG results from an experienced meditator show changes in brain activity in 11 regions shown to be associated with the subjective reports, and these changes occur promptly after jhana is entered. In particular, the extreme joy is associated not only with activation of cortical processes but also with activation of the nucleus accumbens (NAc) in the dopamine/opioid reward system. We test three mechanisms by which the subject might stimulate his own reward system by external means and reject all three. Taken together, these results demonstrate an apparently novel method of self-stimulating a brain reward system using only internal mental processes in a highly trained subject.

## 1. Introduction

Ecstatic experiences have been reported in every major religion, and psychologists have long advocated research in these areas [1, 2]. Neuroscience can contribute to these issues by documenting the brain activity of expert meditators, some of whom have trained to enter these states with volitional control. The type of meditation studied here is a Buddhist concentration technique called jhana that induces an Altered State of Consciousness (ASC) in the framework of Vaitl et al. [3] and whose short-term goal is joy or happiness. Because happiness is a fundamental goal of many people and is the object of the new discipline of positive psychology [4, 5], imaging the brain of an individual who claims to generate joy without any external rewards or cues could point the way toward improved training in joy and greater resilience in the face of external difficulties. Of particular interest is the neural mechanisms by which happiness is generated.

**Figure 1:**
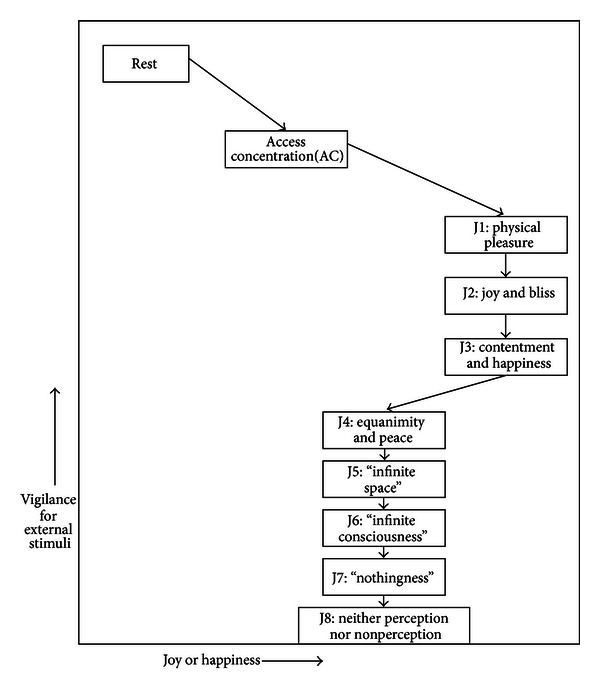
Schematic of the reported experiences in 8 jhanas relative to resting consciousness and access concentration (AC) on 2 dimensions of interest. Joy or happiness is shown on the *x*-axis, and vigilance for external stimuli is plotted on the *y*-axis. The typical meditation sequence is rest to AC to J1, J2, and J3 (the three jhanas highest in joy or happiness), then to J4–J8, all of which are said to be higher in happiness than rest or AC. Each jhana is reported to be deeper and more remote from external stimuli than the last.

Jhana meditations consist of a set of 8 sequential practices that were first codified by Buddhists over 2000 years ago [6]. All are reported to be ecstatic, in that they generate great joy while in an ASC that is dissociated from external cues or stimuli. The first three practices are, to our knowledge, the only meditations to specifically target short-term joy or happiness (see [7, 8] for other meditations that generate ASCs). Figure 1 shows a schematic of the reported jhana experiences on 2 dimensions of interest. Joy or happiness is shown on the *x*-axis, and vigilance for external stimuli is plotted on the *y*-axis. Meditators progress in sequence from normal resting consciousness (rest) to AC, a preparatory meditation concentrating on the breath. When internal concentration is strong enough, J1 is entered, accompanied by strong physical pleasure—“better than sexual orgasm” ([9] p.151)—and greatly reduced vigilance with smaller startle responses. In J2 joy “permeates every part of the body,” but with less physical pleasure. In J3, the character of joy changes to “deep contentment and serenity.” J4 is described by “equanimity—a profound peace and stillness.” The higher-numbered jhanas J5–J8 are characterized by more subtle and profound perceptions. J5 is called “infinite space,” J6 is “infinite consciousness,” J7 is “nothingness,” and J8 is named “neither perception nor non-perception.” Each jhana is reported to be deeper and more remote from external stimuli than the last, yielding the ranking shown on the *y*-axis in Figure 1. J1–J3 are the highest on joy or happiness, with J4–J8 intermediate, yielding the ranking on the *x*-axis. All are classified by Lutz et al. [10, 11] as concentration rather than open awareness meditations.

Previous studies have shown that long-term meditators have higher volume of grey matter compared to matched controls [12, 13], and randomized experiments show that subjects benefit from as little as 4 weeks of training in the areas of attention regulation [14, 15] and emotion regulation [11, 16–18]. Heretofore, all of the emotion studies have tested subjects' ability to learn to downregulate negative emotions, particularly their response to stress. In contrast, the present study examines the ability to up-regulate positive emotion, which involves different neural pathways [19, 20]).

Perhaps the most thoroughly studied system related to positive emotion is the dopamine system, which gives rise to pleasure and mediates positive reinforcement [21, 22]. Both animal and human studies show that when a delivered reward is greater than expected, dopaminergic neurons in the Ventral Tegmental Area (VTA) in the brain stem are activated. The VTA in turn innervates the nucleus accumbens (NAc) in the ventral striatum, which leads to higher centers in the orbital frontal cortex (OFC). Human studies have shown that activity in the medial OFC at the time of a reward correlates with subjective reports of pleasure for olfactory [23], gustatory [24], and musical stimuli [25]. Studies have shown that this system is activated for a diverse array of stimuli, including food [26], sex [27], music [25], humor [28], monetary rewards [29], and maternal love [30]. But it has never been shown that this dopamine system can be activated without external cues or rewards by volitional mental activity. The mechanism by which such a mental activity can self-stimulate positive emotions would be of great interest. One hypothesis is that the full dopamine pathway is stimulated beginning with the VTA and progressing upward. An alternate hypothesis is that the subjective report of pleasure is caused only by expectancy effects (such as a belief that a high-priced wine must taste better; see [31] or [32]) and that the lower parts of the dopamine system do not participate. Yet a third alternative mechanism is that the subjective pleasure is due to subtle rhythmic body movements which are known to induce pleasurable altered states [3].

The dopamine reward system has also been shown to be stimulated by most drugs of abuse and plays an important role in addiction [33]. An important question is whether jhana meditators are subject to addiction and tolerance effects that can result from stimulation of the dopamine reward system. 

Besides the dopamine system, Peciña et al. [34] document that the opioid system mediates pleasure in animal studies. Unfortunately, it shares a pathway very close to that of the dopamine system in the NAc. Discrimination between the two systems would require microinjection studies and is beyond the spatial discrimination of typical fMRI studies. Hence, the current paper limits itself to detecting activation in the region shared by these two reward pathways.

Experientially, all jhanas in Figure 1 are reported to share the following 5 characteristics that may have specific brain correlates: (1) external awareness dims and startle responses diminish, (2) internal verbalizations fade completely or become “wispy”, (3) one's sense of body boundaries and orientation in space are altered, (4) attention is highly focused on the object of meditation, and (5) happiness increases to very high levels and can be maintained for long periods of time. Jhana is distinguished from some other ASCs because it does not include visual or auditory hallucinations (as in some organic disorders and drug experiences) nor does it include cross-sense synesthesia (such as “seeing” the bell ring or “feeling” a bird sing). The correspondences expected from known functions of brain regions can be articulated in the form of the following *a priori* hypotheses.

*H1: Jhanas should show decreased activation compared to the rest state in the visual (BA 17–19) and auditory (BA 41-42) processing areas.* Since all jhanas share the experiential characteristic that external awareness dims, then the brain regions associated with vision and hearing should become less active.

*H2: Jhanas should show decreased activation compared to the rest state in Broca's area (BA 44,45) and in Wernicke's area (BA 39,40).* Because internal verbalization fades in jhana, the brain regions associated with speech should become idle or less active.

*H3: Jhanas should show decreased activation compared to the rest state in the orientation area (BA5).* Since the normal sense of personal boundaries is altered, the orientation area of the brain should show changes from normal rest. Newberg and Iversen [8] showed that monks and nuns experiencing “union with God” exhibit decreased activation in this area.

*H4: Jhanas should show increased activation compared to the rest state in the Anterior Cingulate Cortex (ACC) (BA 32,33).* Because attention is highly focused on the object of meditation in the jhanas, we would expect high activity in the ACC, which regulates and monitors attention.

*H5: Jhanas should show increased activation compared to the rest state in the dopamine reward system of the brain (NAc in the ventral striatum and medial OFC).* A broad range of external rewards stimulate this system (food, sex, beautiful music, and monetary awards), so extreme joy in jhana may be triggered by the same system (the VTA is also part of this system, but is too small to image with standard fMRI methods, but see [35] for successful imaging methods).

*H6: Jhanas should show no increased activation compared to the rest state in the areas responsible for rhythmic movement, including motor cortex (BA4), primary somatosensory cortex (BA 1,2,3), and cerebellum.* Increased activity in these areas would support an alternative hypothesis that the reward system is being stimulated not by internal means but by subtle rhythmic movements that are known to induce ecstatic states [3].

The activation of brain regions during these six subjective jhana experiences can now be examined via fMRI and EEG.

## 2. Methods

The subject is a long-term Buddhist practitioner (53-year-old male, left-handed). At the time of recording, he had 17 years of training consisting of about 6,000 hours of practice and was trained in the Sri Lankan tradition of jhanas by Khema [6] (the length of training was estimated based on his daily practice and the time spent on meditative retreats, counting one day of retreat as 8 hours of sitting meditation). At the time of testing, this subject was to our knowledge the only person in the US who had the requisite training in jhana who was willing to submit to the experimental protocol. The fMRI scanning was done several months after the EEG recording. 

The subject signed informed consent, and a neurological exam was performed, confirming the absence of neurological disease. He had no medical conditions and was on no medications. The subject meditated in his standard sequence, starting with access concentration (AC), progressing through J1, J2,…J8, then returning through J7, J6, and so forth, back down to J1. For each jhana state, the subject signaled with a double finger tap using an MR-compatible force transducer [36] when he was beginning the transition to the next higher-number jhana state, then clicked the mouse once when he had reached the state. He clicked three times to indicate he was transitioning downward to the next lower-number jhana state. Resting periods were recorded before or after jhanas.

 The protocol did not use a random assignment of states because each jhana builds on the previous one, and the time required to transition from one state to another was variable. Hence, the standard sequence was used. This sequence had been very well practiced, making state identification easy for our subject. The duration of each jhana state averaged about 120 sec, with about 30 sec transition between states. 

### 2.1. fMRI Recording and Analysis

We acquired gradient echo T2*-weighted echo-planar images (EPIs) with blood-oxygen-level-dependent (BOLD) contrasts on a GE 1.5-Tesla scanner (repetition time TR of 2.5 s and TE of 50 ms). A total of 421 volumes were collected, with 20 axial slices per volume and slice thickness of 7 mm, going from vertex to inferior cerebellum with no skip between slices. Two T1-weighted structural images were also acquired, the first a high-resolution volumetric series and the second a lower resolution scan in-plane with the functional data. Three periods of rest were interspersed with 2 periods of tapping the force transducer for control purposes, then subject entered AC followed by J2, J3, J4, and J5. The fMRI recording then ended due to scanner memory limitations (421 volume maximum). J1 was not practiced because the associated head movements would induce excessive artifact. 

Statistical parametric mapping [37] served to preprocess and analyze the data. The first four volumes were discarded due to tissue nonsaturation, and each remaining volume was motion corrected to the 5th volume. All images were normalized to a standard MNI template and smoothed using an isometric Gaussian kernel with a full width at half maximum of 8 mm. High-pass filtering was increased to 4096 seconds because the experimental design consisted of a very low frequency of 625 s (from rest to J5). The time signature of the epochs was modeled as a series of boxcar functions convolved with a canonical hemodynamic response function (HRF). The general linear model estimated the percent signal change of each event (jhana versus rest versus AC) as a function of the convolved time signature. The two contrasts of interest in testing the planned hypotheses were jhana-rest and jhana-AC. In addition, J2 was contrasted with each of the other states in order to investigate specific differences between jhana levels of meditation. For each *a priori* ROI specified in the hypotheses, an anatomical mask was prepared from the WFU PickAtlas software [38] and the mean percent signal change was calculated for each contrast using MarsBar [39]. The masks used in this study were Brodmann's area (BA) 17 OR 19, BA 41 OR 42, BA 44 OR 45, BA 39 OR 40, BA 5 OR 7, BA 32 OR 33, BA 1 OR 2 OR 3, and BA 4 (where “OR” refers to the logical addition of two masks), cerebellum, and Med OFC. Finally, the NAc was approximated with spherical masks of radius 5 mm centered at (±10, 9, −4) using the location identified by Kirk et al. [40] and Knutson et al. [41].

### 2.2. EEG Recording and Analysis

The EEG system used a 256-channel Geodesic Sensor Net (System v.2.0 from Electrical Geodesics, OR), sampled at 500 Hz and referenced to the vertex (Cz). Sections of the recording that showed eye movements or muscular artifacts were manually excluded from the study. The data was bandpassed with a digital high-pass filter at .4 Hz and a hardware low-pass filter at 200 Hz. A 60 Hz notch filter was employed to remove 60 Hz line artifacts. Six epochs of 4 seconds each were extracted from each of the 21 states (2 resting states and 19 jhana states).

For each electrode and for each 4 s epoch, the power spectral distribution was computed by using Welch's method, which averages power values across sliding and overlapping 500 ms time windows. Spectral bands were defined to be consistent with previous research: theta band was from 4 to 6 Hz, alpha1 band from 6 to 8 Hz, alpha2 band from 8 to 10 Hz, alpha3 from 10 to 12.5 Hz, beta from 12.5 to 25 Hz, and gamma from 25 to 42 Hz. The last is consistent with Lutz et al. [42] who analyzed only the gamma range. The first 3 bands are congruent with Aftanas et al. [43] who analyzed only those bands. However, we did not perform the analysis of alpha dominant frequency to establish frequency band boundaries individually for our subject, as Aftanas et. al. [43]did, although our band frequencies are close to theirs. All power estimates are reported as a ratio of the power in a selected band to total power from 4 to 42 Hz.

Electrode positions were matched with underlying anatomical ROIs using the probabilistic maps developed by Okamoto et al. [44] who correlated the anatomical MRI's of 17 healthy adults with the overlying electrodes placed in the standard 10–20 position.

## 3. Results

### 3.1. fMRI

Table 1 reports a formal assessment of the 6 *a priori* hypotheses. The first row of Table 1 tests H1, where the first column shows the subjective experience during jhana (that external awareness dims), the second column shows the ROI associated with that experience (the primary and associative visual cortex BA 17,19), and the third column shows predicted change in activity during jhana compared to rest (activity will be less during jhana). Column 4 shows that the actual contrast is −.81, a difference that is significant (*t* = −4.3, *P* < .001) and in the predicted direction. The last column of Table 1 uses an alternative comparison standard, calculating the BOLD signal contrast for Jhana relative to access concentration (Jhana-AC). That column confirms that the contrast is also negative, supporting the reports in Figure 1. The next row shows that the contrast in primary auditory and association cortex (BA 41, 42) was also negative and significant, again supporting H1. Similarly, H2 (that internal verbalization fades) is strongly supported by significant negative contrasts in Broca's area (BA 44, 45) and in Wernicke's area (BA 39, 40). H3 (an altered sense of personal boundaries) is strongly supported with large and significant negative signal contrasts in the orientation area (BA 5, 7). H4 (that attention is highly focused) is more weakly confirmed, with both BOLD signal contrasts in the ACC positive compared to rest, though column 5 shows that the contrast Jhana-AC failed to reach significance. H5 is strongly confirmed, with both the NAc and Med OFC recording significantly higher BOLD signal during jhana than during both rest and AC meditation. The last rows of Table 1 show the test of an alternative hypothesis (H6) that the ecstatic joy in jhanas may be caused by subtle rhythmic movements, resulting in higher BOLD signal during jhana in the primary somatosensory cortex, the primary motor cortex, and the cerebellum. This alternative hypothesis was strongly rejected in all 3 areas.

**Figure 2:**
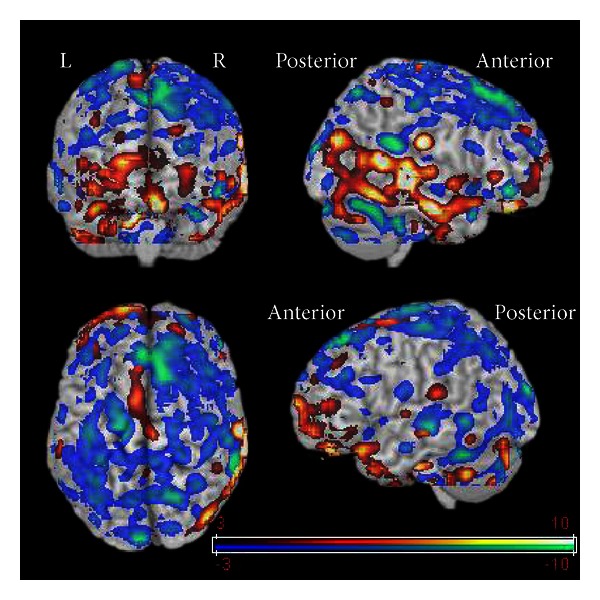
Cortical surfaces with post hoc *t* values greater than +3 (in red and yellow) or −3 (in blue and green) as calculated by SPM5 using the BOLD contrast (jhana-rest). Note that transition to jhana is associated with selective increases in BOLD signal in right temporal region and with decreases in parietal lobe and posterior frontal lobe.

In addition to testing the six *a priori* hypotheses, standard SPM5 statistical tests using post hoc analysis were computed for all brain tissue. Figure 2 displays all cortical surfaces with post hoc *t* values greater than +3 (in red and yellow) or −3 (in blue and green) in the contrast (jhana-rest). It shows very extensive but “patchy” areas of activation, with 63 clusters significantly positive, and 27 clusters were significantly negative, suggesting an overall pattern of diffuse activation during jhana. Perhaps the most evident results in Figure 2 are that transition to jhana is associated with selective decreases in BOLD signal in the parietal and posterior frontal lobes (confirmed by *a priori* tests above) and with selective increases in the right temporal region. 

**Figure 3:**
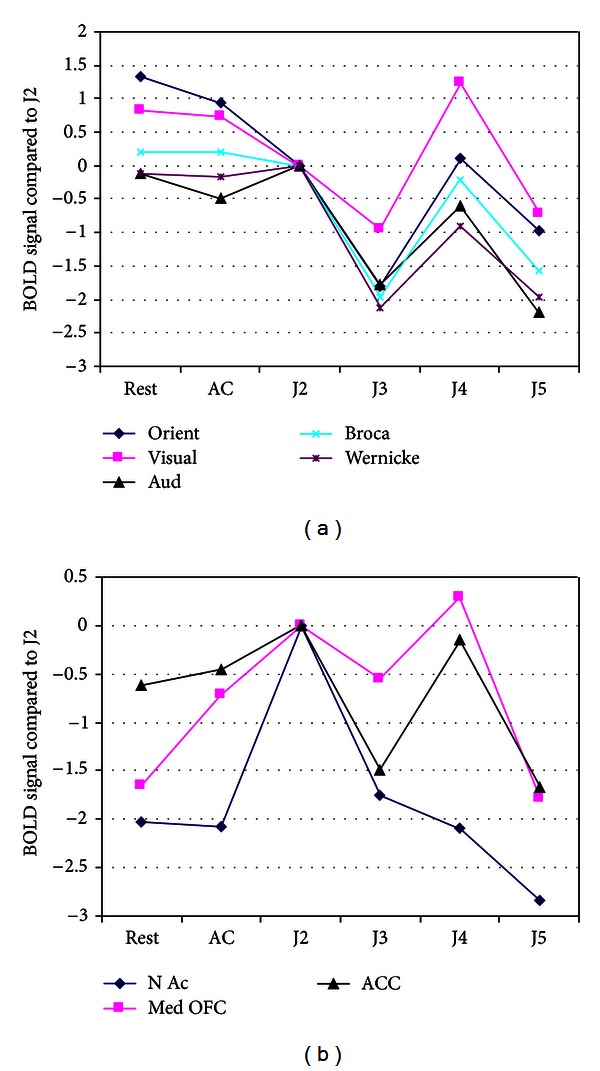
Average BOLD signal of each meditation state contrasted with J2 is shown on the *y*-axis, with a separate line for each ROI. The *x*-axis denotes each state from rest to AC to J2–J5. The mean SE of the signal contrasts averaged over the ROIs and states was ± .3. Note the high correlation between the lines in (a) and the steep increase in “NAc” at J2 in (b) (J1 was not recorded due to head movement artifacts).

Given that the data support the six hypotheses, we then disaggregated the results to explore whether the different jhana meditation states produced different brain activation patterns. Figure 3(a) plots the BOLD signal of each state contrasted with J2, with a separate line for each of the ROIs from H1 to H3. For example, the line labeled “orientation” plots the BOLD signal (relative to J2) on the *y*-axis as a function of meditation state on the *x*-axis, progressing from rest to AC to J2 and on through J5. It shows a steep decline from rest and AC to J2, and another steep decline to J3, then reaches a global maximum at J4, followed by a return to the low levels of J3. Interestingly, the remaining four lines in Figure 3(a) are highly correlated with the “orientation” line, showing similar patterns of decline, steep increases at J4, and return to low values at J5. The correlation suggests an association between the ROIs, the most likely being that reduced activation of vision and audition will “deafferent” the orientation area from its normal inputs, causing an altered sense of orientation. 

Figure 3(a) also gives a more nuanced view of individual jhanas than the pooled results in Table 1. While the average jhana shows lower activation than rest and AC (as predicted by H1–H3), the individual jhanas show great variability, with lower activation in J2, J3, and J5, (as predicted by traditional reports in Figure 1), but J4 shows activation equal to or higher than rest. We caution that this figure plots single meditation states of an individual, so that a single distractor event could greatly alter the activation pattern during a meditation. In this case, a distractor event may have occurred during J4, causing increased activity in visual, auditory, and orientation area (however, the subject did not report any distractions during debriefing). A final deviation from predictions is that no decline in activation occurs after J2, whereas Figure 1 would predict that activity will decline with each successive jhana in areas associated with sensing external stimuli. 

Figure 3(b) plots the BOLD contrast (relative to J2) of the remaining ROIs as a function of meditation state on the *x*-axis, progressing from rest through J5. The line denoted as “NAc,” shows a very steep increase in activation from rest and AC to J2, consistent with Figure 1. But activity in the NAc declines during J3 to near that of rest and AC and declines even further in J5, consistent with a dopamine depletion hypothesis in later jhanas. The line for Med OFC shows moderate decline during J3 and reaches its maximum at J4. This pattern contrasts with the predictions of Figure 1 where J4 is reported as less joyful than J2 and J3. Finally, the line labeled “ACC” shows increased monitoring from rest to J2, declining to lower monitoring at J3 and J5, but spiking at J4. Since Figure 3(b) shows that J2 was the only jhana to activate the complete dopamine pathway, tests of the alternative hypothesis were conducted on J2 alone. Consistent with the pooled results in Table 1, the alternative hypothesis (H6) that subtle rhythmic movements triggered joy in J2 was rejected, with significantly lower activity in areas associated with movement during J2 compared to rest in BA 1,2,3 (*t* = −4.7, *P* < .001), BA 4 (*t* = −4.5, *P* < .001), and in the cerebellum (*t* = −1.75 n.s.). All signs were in the opposite direction from that predicted by the alternative hypothesis. 

**Figure 4:**
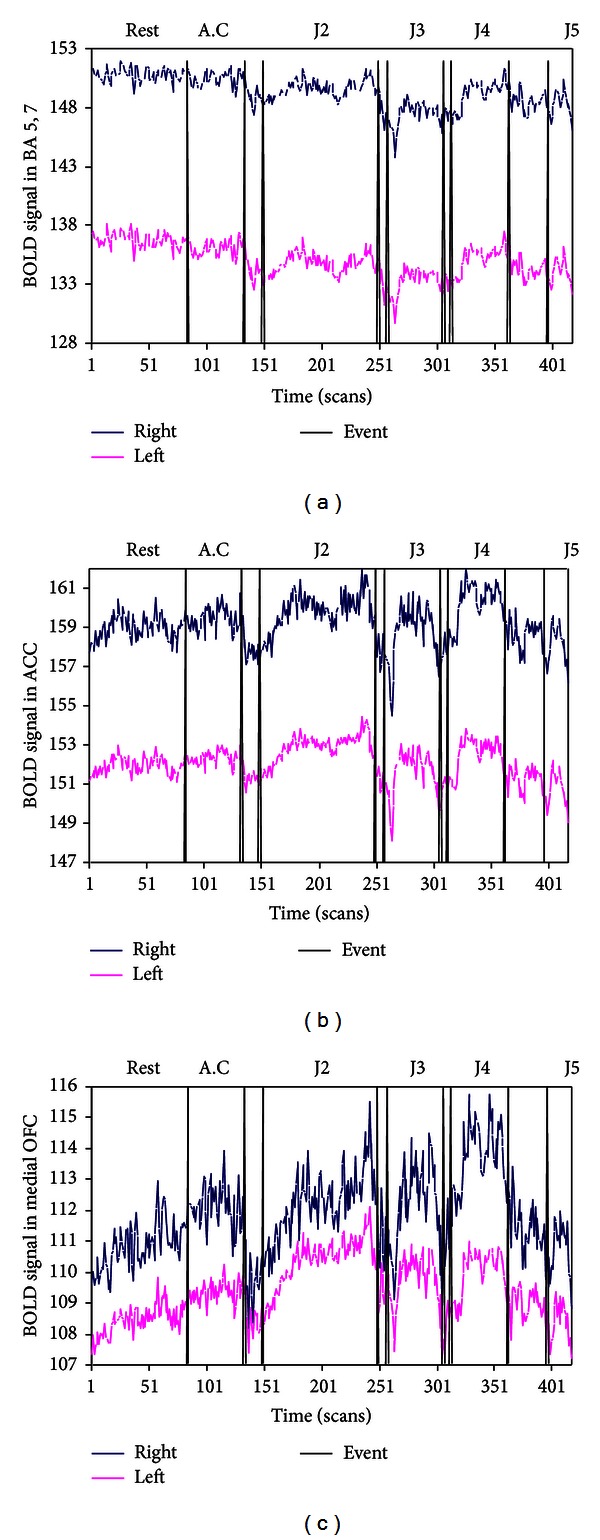
Time course (in fMRI scans) of BOLD signal for three *a priori* defined ROIs (blue line shows right side of ROI, and red line shows right side) graphing transitions between rest, access concentration, and jhanas. Figure 4(a) shows BOLD signal averaged for all voxels in BA 5 and 7 (orientation area), Figure 4(b) shows average BOLD in ACC, and Figure 4(c) shows average BOLD in medial OFC. Note the prompt drop in signal during transition events, including the decline in BA 5,7 activity during jhanas and the increase in OFC signal during jhanas (J1 was not recorded due to head movement artifacts).

Figure 4 shows more detailed dynamics of the state transitions, with the time course of the BOLD signal averaged over all voxels in three *a priori* specified ROIs during the 417 fMRI scans.  Figure 4(a) shows average BOLD signal for the orientation area BA 5 and 7, with the blue line representing the right side and the red line representing the left. The black spikes extending from the *x*-axis represent events where the meditator signaled a transition to a higher state with a mouse click. Note the steep drop during the transitions from AC to J2 and J2 to J3. These drops are not caused by the clicking action because they do not appear during transition from rest to AC. The drops occurred promptly after the subject signaled that he was starting to transition, beginning within 2 scans (5 sec) and reaching minimum within 8 scans (20 sec) during the AC to J2 transition, with similarly prompt transitions from J2 to J3. Figure 4(b) shows the BOLD signal in the right and left ACC regions, with similar steep and prompt drops during the transitions from AC to J2, J2 to J3, and J4 to J5. Finally, Figure 4(c) shows the BOLD signal in the right and left medial OFC, with even steeper drops during the transitions from AC to J2, J2 to J3, J3 to J4, and J4 to J5.

### 3.2. EEG Results

The EEG data were first examined for outliers and missing data. There were no bad channels, so spatial interpolation was not required. Though no missing data was found, all of the data for J1 are outliers, with putative gamma power at least 10 times the gamma power of other jhanas and rest. It is likely that much of the gamma was due to muscle tension because of head movements. Hence J1 is excluded from analysis because it was more than 4 standard deviations away from any other state. All data for remaining states were approximately normally distributed.

Statistical tests for the planned comparisons that test H1–H6 are presented in Table 2. Similar to Table 1, column 1 shows the subjective experience, column 2 shows the ROIs and the scalp electrode locations (from [44]) associated with that experience, and column 3 shows the predicted direction of contrasts between jhana and rest. Column 4 shows the actual gamma power (25–42 Hz) measured at that scalp location. In the case of the first row, the gamma power at O1 (overlying the primary and associative visual cortex BA17, 19) showed no significant difference between jhana and rest. Examining all rows of column 4 shows that gamma power increased significantly only in the electrode locations overlying the ACC and the Med OFC, consistent with H4 and H5. However, in locations overlying regions expected to decrease activation (H1, H2, H3, and H6), all showed nonsignificant contrasts in the gamma range (with the exception of C4, which was in the predicted direction). We also examined contrasts in the alpha1 range (not shown), which Laufs et al. [45] demonstrated are negatively correlated with fMRI activation. Twelve of the 14 contrasts testing H1, H2, H3, and H6 showed significant increases in the alpha1 range, consistent with the hypotheses. We integrated the power information from many bands in column 5, which calculates the difference in power between the high frequencies (gamma + beta) minus the power in the lower frequencies (alpha1 + theta) for jhana compared to rest. Consistent with column 4, the largest increases in activation during jhana are observed near the Med OFC (H5), accompanied by smaller but very significant increases in ACC (H4). Significant declines in activity during jhana are observed near BA 17,19, BA 41,42, BA 44,45, BA 5,7, and BA 1,2,3, consistent with those hypotheses (H1, H2, H3, and H6).

## 4. Discussion

The fMRI and EEG recordings provide mutually consistent evidence on the neural correlates of ecstatic meditations called jhanas. In the cortical regions associated with external awareness, verbalization, and orientation (H1, H2, and H3), Table 1 shows a lower fMRI BOLD signal during jhana contrasted with rest. In addition, Table 2 shows that the EEG signal shifted to the lower-power bands of theta and alpha1, although it is acknowledged that spatial localization of cortical function with scalp EEG has some limitations. In the region associated with executive control (H4) and the region associated with subjective happiness (H5), the fMRI in Table 1 showed higher BOLD signal during jhana contrasted with rest, while the EEG in Table 2 showed a shift to higher power in the beta and gamma bands. In addition, the subcortical imaging from the fMRI was able to distinguish whether the subjective happiness (H5) was associated with activation of the dopamine/opioid reward system or due to purely cortical expectation effects. Table 1 (in the row H5) shows very strong activation of the NAc in the ventral striatum indicating that the full pathway was activated in at least one of the jhanas. 

Examining individual jhanas revealed several deviations from the predictions derived from subjective reports in Figure 1. First, activity in orientation and visual areas does decline below rest and AC but does not decline further after J3, contrary to reports that each succeeding jhana goes deeper. Second, activity in the NAc peaks during J2 and then drops quickly, contrary to reports that J3 is equally joyful. We conclude that full activation of the dopamine reward system occurred only in J2, while J3 activated only the Med OFC portion of the reward system.

Previous imaging of the dopamine/opioid reward system has always used external stimuli to activate it (e.g., actual food or drink was consumed or photos of loved ones cued a short period of attachment). In contrast, jhana meditators claim that they can voluntarily generate increased happiness purely by volitional mental processes and for extended periods. We tested this claim in several ways. First, we examined ROIs associated with somatosensory and motor coordination, which would be active if the subject was making subtle rhythmic movements known to trigger ecstatic ASCs [3]. These areas were not found to show increases but instead showed significant decreases in activity during J2, consistent with the claim that the reward system is triggered without physical cues or imagined movements. Another alternative hypothesis is that the subject was using indirect mental processes to stimulate the reward system such as evoking a visual or auditory memory of a happy time, which in turn would trigger the reward system. However, our evidence in Tables 1 and 2 (row H1) showed that the cortical ROIs associated with vision and hearing declined significantly in activity during jhana (and Figure 3(a) confirms this specifically for J2), contrary to this alternate hypothesis. Finally, evidence on lateralized brain activation such as those related to H2 and Wernicke's area must be interpreted with some caution, as the subject examined in the current study was left handed. Left handedness can be associated with structural and functional changes in brain symmetry, as compared to the majority of human subjects, who are strongly right handed [46, 47], and this fact might have influenced some results in Figures 3 and 4.

### 4.1. Mechanisms of Action

Our data would reject four possible cortical mechanisms (expectations, rhythmic movement, visual memories, and auditory memories) by which the subject might have self-stimulated his own reward system during J2. Several other pathways are possible that our experiment did not test. First, it is known that reciprocal connections exist between the NAc and the medial OFC, so that it might be possible to activate a feedback loop between the two. Under normal conditions, the feedback loop would be quickly interrupted by shifting attention to everchanging input from visual, auditory, or somatic senses, but these cortical areas have been downregulated, and attention may be tightly focused on reinforcing the feedback loop. The loop might be realized by creating a series of very short tasks that can each be completed successfully, allowing a new goal to be achieved and reward attained with each new moment. The classic meditation instructions for breathing would constitute such a task, wherein the student is instructed: “When that in-breath finishes, you know that moment. You see in your mind that last moment of the in-breath. You then see the next moment as a pause between breaths, and then many more moments of pause until the out-breath begins… We are aware only of the beautiful breath, without effort and for a very long time.” ([9] p.16).

Other possible mechanisms of action could comprise subcortical activations that might have reward characteristics. For example, shifting control of breathing from the voluntary motor cortex to the involuntary medullary rhythmicity area in the brain stem might be perceived as relaxing, as well as giving rise to a common altered experience of “feeling like I am being breathed, not in control.” Also, rhythmic movements might be maintained below the level of cortical control, since spinal reflexes are now known to mediate rhythmic movements as complex as coordinating leg movements related to walking. 

Our results also shed light on the *magnitude* of the activation of the dopamine reward system. Subjective reports from the subject indicated extremely high magnitude of reward, comparing J1 (which was not recorded due to head movement) to continuous multiple orgasms, J2 to “opening a birthday gift and getting exactly what you most wished for,” and J3 to postcoital bliss. Yet the objective activation of the reward system in J2 was not extreme. The apparent mismatch between extreme subjective reports and moderate objective activation can be explained by the signal-to-noise ratio of the circuits. When most other cortical activity is reduced, as in this subject, a much smaller reward signal can be detected and will be perceived as more intense than when cortical “noise” from other sources is high, as in normal awareness. Indeed, during normal awareness it takes drug-induced hyperstimulation of the dopamine pathways to generate such extreme subjective reports. If this signal-to-noise view is correct, then jhana's reduced sense awareness is not incidental to achieving extreme pleasure but is a contributing condition. 

Despite the moderate level of activation, caution is advisable with any voluntary stimulation of the reward systems. Drugs of abuse can generate short-term bliss but can quickly increase tolerance, requiring ever greater doses of the drug to create the same level of pleasure. They can also create withdrawal symptoms during abstinence [33]. In contrast to the drugs, jhana meditators report negative tolerance because they can achieve the same state more quickly with less effort over time, and no withdrawal symptoms have been reported when meditation is stopped. Nevertheless, Figure 3 shows that NAc activity dropped below normal resting consciousness in J5, which may be a sign of short-term tolerance and neurotransmitter depletion.

### 4.2. Implications

Our experiment is to our knowledge the first that compares brain states in five different meditations (AC and J2–J5), finding strong differences between AC meditation and jhana, and smaller but still significant differences between jhana states. These in turn differ from the Tibetan Buddhist compassion meditation reported by Lutz et al. [42] where EEG gamma frequencies were dominant and from the alpha dominance of Transcendental Meditation [48]. Taken together, the multiplicity of brain states suggests that there may be a vast array of ASCs available through meditation, depending on which brain regions are given awareness and which are inhibited from awareness [49]. If there are a large number of possible ASCs, it is likely that only some would have survival value. For example, the state of mystical union with all beings might be helpful in encouraging cooperation with all people in the tribe, so that evolution may have selected certain of these ASCs to be more easily learned and retained. 

However, the same reasoning would suggest that the ability to self-stimulate the brain's reward system would be dysfunctional in the struggle for survival and procreation because it could short-circuit the system that motivates survival actions. Organisms that are adept at self-stimulation would quickly die out if they fail to respond to environmental demands or to pass on their genes. This reasoning suggests caution in making autonomous self-stimulation more available, but we point out that the modern environment already allows unprecedented stimulation of the dopamine reward system with plentiful food and drugs of abuse. A meditation that stimulates the reward system without the harmful effects of obesity and environmental damage could be beneficial in the modern environment. On the other hand, a meditation that short-circuits the desire to get an education and work for long-term goals could become dysfunctional. Rather than simply *stimulating* the reward system in response to traditional goals of food and sex, it would be beneficial to *regulate* the system and focus it on long-term goals that are more adaptive.

This case study provides guidelines for larger studies on jhana meditation in several areas. First, it demonstrates that jhana is not so fragile that it can be destroyed by the presence of curious experimenters or by intrusive sounds of MRI scanners. Hence, it can be scientifically investigated. Second, the transition time to move from one jhana to another in a practiced subject is much shorter (between 5 and 20 seconds) than we expected, in line with other meditations that do not produce such extreme ASCs [42]. With short transition times, it might be feasible to use better randomized designs that alternate control states with meditations (however, the short transition times here may be due to the subject's internal knowledge of readiness to transition, and he may not be able to transit “on demand”). Third, the experiment could be shortened if interest is focused only on the reward system because only J2 shows strong self-activation of the NAc. Fourth, the simple “resting” condition used here could be replaced with better controls that have been demonstrated to increase happiness, such as “remembering a happy event in your life” or visualizing a loved one.

More potential subjects will become available as more English-speaking students are being trained in jhana meditation [9, 50]. How these meditators achieve periods of extreme joy without common negative side effects could contribute to the scientific “pursuit of happiness” and could pave the way for novel paradigms for rehabilitation and recovery from nervous system injury.

## Figures and Tables

**Table 1:** Mean percent BOLD signal change in *a priori* defined ROIs related to 6 hypotheses on jhana activity contrasted with rest and AC meditation, followed by its two-sided *t* test (corrected for multiple comparisons). Contrasts labeled simply “jhana” refer to the pooled activity over all recorded jhanas 2–5. All 22 planned contrasts are in the predicted direction.

Subjective report during jhanas	*A priori* ROI (MNI coordinates of centroid of ROI)	Predicted sign of contrast (jhana-rest)	BOLD contrast (jhana-rest)	BOLD contrast (jhana-AC)
(1) “External awareness dims”	Visual: BA 17, 19 (±30 −80 6)	(−)	−.81 *t* = −4.3**	−.73 *t* = −4.0**
	Auditory: BA 41, 42 (±55 −26 12)	(−)	−.63 *t* = −2.5*	−.25 *t* = −1.0

(2) “Internal verbalization fades”	Broca: BA 44, 45 (±54 18 12)	(−)	−.84 *t* = −4.6**	−.85 *t* = −4.8**
	Wernicke: BA 39, 40 (±51 −51 34)	(−)	−.76 *t* = −3.7**	−.70 *t* = −3.5**

(3) “Altered sense of personal boundaries”	Orientation: BA 5, 7 (±17 −59 52)	(−)	−1.8 *t* = −6.9**	−1.4 *t* = −5.6**

(4) “Attention is highly focused”	ACC: BA 32, 33 (±8 36 14)	(+)	.62 *t* = 2.86*	.10 *t* = .44

(5) “Ecstatic joy experienced”	N Ac (±10 9 −4)	(+)	.88 *t* = 3.5**	.94 *t* = 3.8**
	Med OFC (±8 50 −9)	(+)	1.44 *t* = 7.2**	.49 *t* = 2.6*

(6) Less rhythmic movement	Somatosens: BA 1, 2, 3 (±39 −28 53)	(−)	−1.50 *t* = −7.3**	−1.38 *t* = −6.9**
	Prim Motor: BA 4 (±35 −23 53)	(−)	−1.47 *t* = −5.8**	−1.38 *t* = −5.6**
	Cerebellum (±0 −61 −34)	(−)	−.77 *t* = 4.3**	−.62 *t* = −3.6**

**P < .001.

*P < .05.

BA: Brodmann's area, NAc: nucleus accumbens, Med OFC: medial orbitofrontal cortex, and ACC: anterior cingulate cortex.

**Table 2:** Contrasts in the spectral power of the EEG signal in selected bands at *a priori* defined scalp locations related to 6 hypotheses on jhana activity compared with rest, followed by its *F*-test on the null hypothesis that jhana activity is equal to rest activity. All *F* statistics are with degrees of freedom of (1,502). Contrasts labeled simply “jhana” refer to the pooled activity over all recorded jhanas 2–5. In the last (summary) column, all significant differences are in the direction predicted by the 6 hypotheses.

Subjective report during jhanas	*A priori* ROI (scalp electrode locations)	Predicted sign of contrast (jhana-rest)	Contrast of power in gamma range (jhana-rest)	Contrast in power of (gamma + beta) − (alpha1 + theta)
(1) “External awareness dims”	Visual: BA 17, 19			
	(O1)	(−)	Ns	−.08 *F* = 5.6*
	(O2)		Ns	−.11 *F* = 8.3*
	Auditory: BA 41, 42			
	(T3)	(−)	Ns	Ns
	(T4)		Ns	−.10 *F* = 15**

(2) “Internal verbalization fades”	Broca: BA 44, 45			
	(FC5)	(−)	Ns	−.07 *F* = 9.3*
	Wernicke: BA 39, 40			
	(Tp7)	(−)	Ns	Ns

(3) “Altered sense of personal boundaries”	Orientation: BA 5, 7			
	(P1)	(−)	Ns	−.08 *F* = 6.6*
	(P2)		Ns	−.12 *F* = 13**
	(P3)		Ns	−.06 *F* = 5.0*
	(P4)		Ns	−.11 *F* = 16**

(4) “Attention is highly focused”	ACC: BA 32, 33			
	(AFz)	(+)	+.030 *F* = 21**	+.12 *F* = 27**
	(Fz)		+.014 *F* = 8.0*	+.07 *F* = 7.8*
	(FCz)		−.015 *F* = 5.1*	Ns

(5) “Ecstatic joy experienced”	N Ac	(+)	(unobservable)	(unobservable)
	Med OFC			
	(Fp1)	(+)	+.104 *F* = 57**	+.42 *F* = 149**
	(Fp2)		+.092 *F* = 38**	+.35 *F* = 75**

(6) Less rhythmic movement	Somatosens: BA 1, 2, 3			
	(C3)	(−)	Ns	−.07 *F* = 8.7*
	(C4)		−.013 *F* = 4.6*	−.11 *F* = 15**
	Prim motor: BA 4			
	(FC3)	(−)	Ns	Ns
	(FC4)		Ns	Ns
	Cerebellum	(−)	(unobservable)	(unobservable)

*P < .05.

**P < .001.

BA: Brodmann's area, NAc: nucleus accumbens, Med OFC: medial orbitofrontal cortex, and ACC: Anterior cingulate cortex.
